# Analysis of BRCA1/2 variants of unknown significance in the prospective Korean Hereditary Breast Cancer study

**DOI:** 10.1038/s41598-021-87792-w

**Published:** 2021-04-19

**Authors:** Joo Heung Kim, Sunggyun Park, Hyung Seok Park, Ji Soo Park, Seung-Tae Lee, Sung-Won Kim, Jong Won Lee, Min Hyuk Lee, Sue K. Park, Woo-Chul Noh, Doo Ho Choi, Wonshik Han, Sung Hoo Jung

**Affiliations:** 1 Department of Surgery, Yongin Severance Hospital, Yonsei University College of Medicine, Yongin, Gyeonggi Republic of Korea; 2 Department of Laboratory Medicine, Keimyung University School of Medicine, Daegu, Republic of Korea; 3 Department of Surgery, Yonsei University College of Medicine, 50-1 Yonseiro, Seodaemun-gu, Seoul, 03722 Republic of Korea; 4 Hereditary Cancer Clinic, Cancer Prevention Center, Yonsei Cancer Center, Yonsei University College of Medicine, 50-1 Yonseiro, Seodaemun-gu, Seoul, 03722 Republic of Korea; 5 Department of Laboratory Medicine, Yonsei University College of Medicine, Seoul, Republic of Korea; 6 Department of Surgery, Daerim St. Mary’s Hospital, Seoul, Republic of Korea; 7 Department of Surgery, Asan Medical Center, University of Ulsan College of Medicine, Seoul, Republic of Korea; 8 Department of Surgery, Soonchunhyang University Seoul Hospital, Seoul, Republic of Korea; 9 Department of Preventive Medicine, Seoul National University College of Medicine, Seoul, Republic of Korea; 10 Cancer Research Institute, Seoul National University, Seoul, Republic of Korea; 11 Department of Surgery, Korea Institute of Radiological & Medical Science, Korea Cancer Center Hospital, Seoul, Republic of Korea; 12 Department of Radiation Oncology, Samsung Medical Center, Sungkyunkwan University, Seoul, Republic of Korea; 13 Department of Surgery, Cancer Research Institute, Seoul National University College of Medicine, Seoul, Republic of Korea; 14 Department of Surgery, Chonbuk National University Hospital, Jeonju, Jeollabuk Republic of Korea

**Keywords:** Cancer, Genetics, Medical research

## Abstract

Genetic testing for *BRCA1* and *BRCA2* is crucial in diagnosing hereditary breast and ovarian cancer syndromes and has increased with the development of multigene panel tests. However, results classified as variants of uncertain significance (VUS) present challenges to clinicians in attempting to choose an appropriate management plans. We reviewed a total of 676 breast cancer patients included in the Korean Hereditary Breast Cancer (KOHBRA) study with a VUS on *BRCA* mutation tests between November 2007 and April 2013. These results were compared to the ClinVar database. We calculated the incidence and odds ratios for these variants using the Korean Reference Genome Database. A total of 58 and 91 distinct VUS in *BRCA1* and *BRCA2* were identified in the KOHBRA study (comprising 278 and 453 patients, respectively). A total of 27 variants in the KOHBRA study were not registered in the Single Nucleotide Polymorphism database. Among *BRCA1* VUSs, 20 were reclassified as benign or likely benign, four were reclassified as pathogenic or likely pathogenic, and eight remained as VUSs according to the ClinVar database. Of the *BRCA2* VUSs, 25 were reclassified as benign or likely benign, two were reclassified as pathogenic or likely pathogenic, and 33 remained as VUS according to the ClinVar database. There were 12 variants with conflicting interpretations of pathogenicity for *BRCA1* and 18 for *BRCA2*. Among them, p.Leu1780Pro showed a particularly high odds ratio. Six pathogenic variants and one conflicting variant identified using ClinVar could be reclassified as pathogenic variants in this study. Using updated ClinVar information and calculating odds ratios can be helpful when reclassifying VUSs in *BRCA1/2*.

## Introduction

Hereditary breast and ovarian cancer syndrome (HBOC) has been shown to be associated with germline mutations in *BRCA1* and *BRCA2*^1^, spurring demands for genetic testing to identify pathogenic variations in these genes^2^. The identification of a pathogenic BRCA mutation in a patient diagnosed with breast cancer not only affects their treatment and prognosis, but also enables the prevention of other cancers^3^. Guidelines for the management of pathogenic variants in *BRCA1* and *BRCA2* recommend consideration of risk-reducing medications or surgeries^4,5^.

A genetic test for *BRCA* has four possible results: no mutation detected, pathogenic mutation, benign mutation, or variant of uncertain significance (VUS). A VUS is an alteration in the gene sequence that has an unknown effect on the function of the gene product. This leaves patients and their physicians with uncertainty due to the inability to interpret the result in a clinical context and a lack of specific guidelines regarding genetic counseling or prophylactic management in mutation carriers and their relatives^6^.

While an overall VUS rate of 7–15% in women who have received *BRCA* testing has been reported^7^, the frequency of VUS varies worldwide depending on the testing prevalence and population ancestry^7,8^. Researchers reported a frequency of VUS of 21% in African-Americans, 5–6% in people of European ancestry in the United States, and 15% in European laboratories^9,10^. Myriad Genetic Inc. (Salt Lake City, UT, USA) reported that they decreased the proportion of VUSs to 2.1% using accumulated data^11^. However, these databases are not public or accessible.

In this study, we aimed to explore the prevalence of VUS in the Korean population and to reclassify these variants using the ClinVar database and the Korean Reference Genome Database (KRGDB).

## Results

### Baseline characteristics

**Figure 1:**
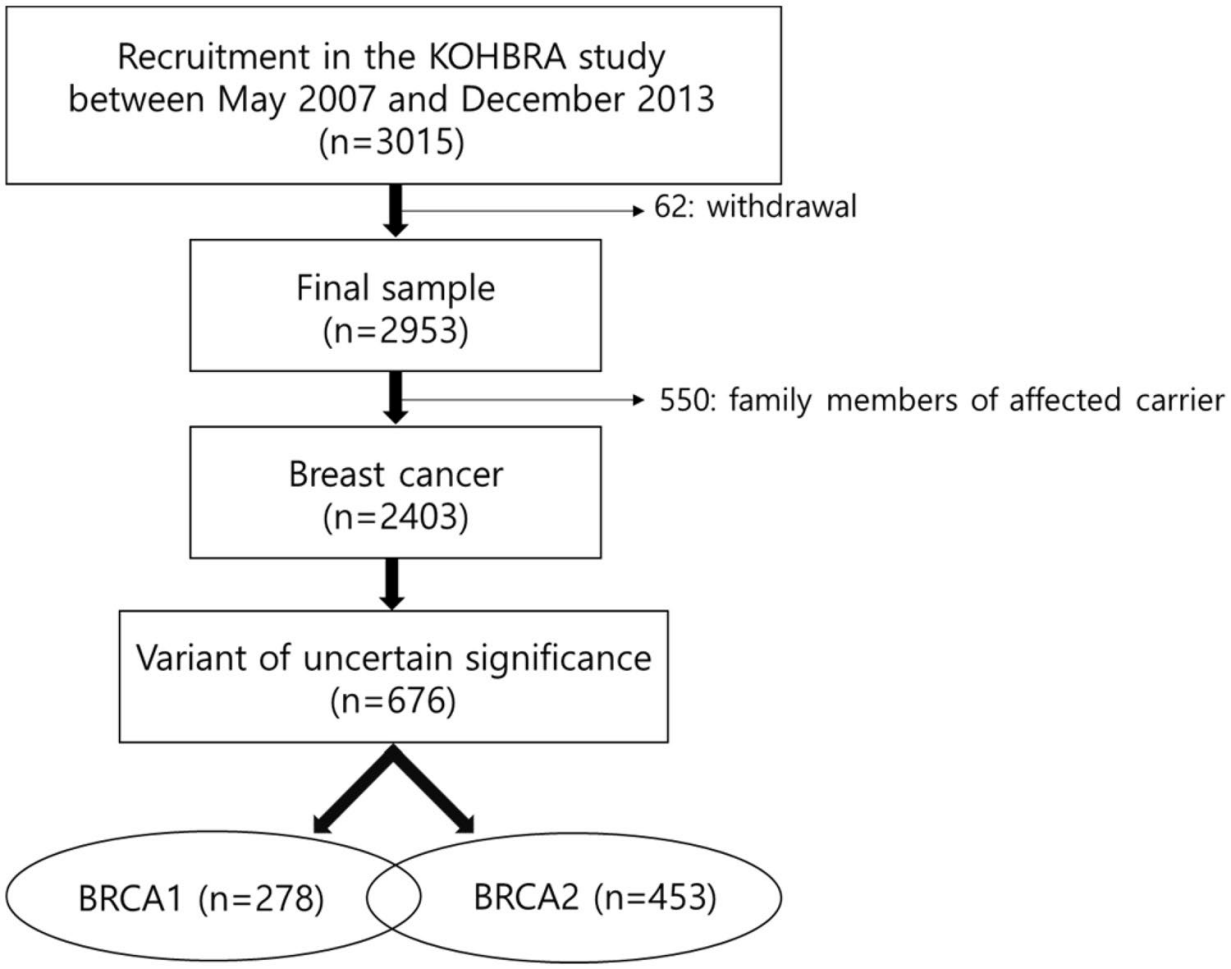
Schematic diagram of patient selection: BRCA1 and BRCA 2 (n = 55).

Among 2,403 breast cancer patients in the Korean Hereditary Breast Cancer (KOHBRA) study, more than a quarter, 676 (28.13%) patients, had mutations that were classified as VUS. Simultaneous mutations of *BRCA1* and *BRCA2* were observed in 55 (55/676, 8.14%) patients. Of the 676 subjects, 278 had a VUS in *BRCA1*, and 453 patients had a VUS in *BRCA2* (Fig. 1). refSNP (RS) numbers were reviewed for 262 and 440 subjects with *BRCA1* and *BRCA2* mutations, respectively.

### Reclassification using public databases

Table 1 shows the reclassification results for VUS according to the ClinVar database. We classified the results into four groups: benign/likely benign, VUS, conflicting interpretations of pathogenicity, and pathogenic/likely pathogenic. Benign/likely benign was the most common reclassification for both *BRCA1* and *BRCA2* VUSs. Table 2 shows the rank of VUS genes based on the number of patients.

**Table 1:** Reclassification of patients diagnosed with variants of uncertain significance based on ClinVar data.

BRCA1	58 mutations (n = 278)
Benign/ likely benign	20 mutations (n = 193)
VUS	8 mutations (n = 8)
Conflicting in interpretations of pathogenicity	12 mutations (n = 55)
Pathogenic/ likely pathogenic	4 mutations (n = 6)
Variants not registered in SNP database	14 mutations (n = 16)

BRCA2	91 mutations (n = 453)
Benign/ likely benign	25 mutations (n = 328)
VUS	33 mutations (n = 58)
Conflicting in interpretations of pathogenicity	18 mutations (n = 51)
Pathogenic/ likely pathogenic	2 mutations (n = 3)
Variants not registered in SNP database	13 mutations (n = 13)

**Table 2:** Top 10 high-frequency mutations based on the number of patients.

BRCA1	RS_number	ClinVar	No. of patients	OR	CI
c.4883T>C	rs4986854	Benign	57	0.8649	0.5865–1.2754
c.4484 + 14A>G	rs80358022	Benign	41	0.5823	0.3845–0.8818
c.2566T>C	rs80356892	Benign	40	0.3659	0.2491–0.5374
c.3113A>G	rs16941	Benign	17	0.0079	0.0049–0.0128
c.154C>T	rs80357084	Conflicting interpretations of pathogenicity	15	0.4646	0.2421–0.8914
c.5339T>C	rs80357474	Conflicting interpretations of pathogenicity	12	8.5181	1.1192–64.8277
c.4729T>C	rs80356909	Conflicting interpretations of pathogenicity	9	1.2874	0.4314–3.8417
c.3448C>T	rs80357272	Benign	8	0.6349	0.2448–1.6464
c.3548A>G	rs16942	Benign	8	0.0046	0.0025–0.0086
c.547 + 14delG	rs273902771	Conflicting interpretations of pathogenicity	6		

BRCA2	RS_number	ClinVar	No. of patients	OR	CI
c.10234A>G	rs1801426	Benign	67	0.6443	0.4627–0.8972
c.8187G>T	rs80359065	Benign	57	0.7962	0.5445–1.1643
c.9649-19G>A	rs11571830	Benign	40	0.5462	0.3609–0.8266
c.5785A>G	rs79538375	Benign	33	0.8118	0.4922–1.339
c.2971A>G	rs1799944	Benign	29	0.0432	0.0299–0.0623
c.2350A>G	rs11571653	Benign	28	0.4624	0.2868–0.7456
c.1744A>C	rs80358457	Benign	24	0.7135	0.4046–1.2581
c.7052C>G	rs80358932	Benign/likely benign	12	0.6115	0.2826–1.3231
c.6325G>A	rs79456940	Conflicting interpretations of pathogenicity	10	0.6488	0.2754–1.5285
c.623T>G	rs80358865	Uncertain significance	9	1.2867	0.4313–3.8389

Of the 278 patients with a *BRCA1* VUS, 58 VUSs were identified, and 44 had RS numbers. Twenty of these variants, found in 193 patients, were classified as benign/likely benign. The least common VUSs were classified as pathogenic/likely pathogenic and comprised four variants in six patients (Table 1).

Of the 453 patients with *BRCA2* VUSs, 91 VUSs were identified, and 78 had RS numbers. The most common VUSs were benign/likely benign, comprising 25 variants in 328 patients. Meanwhile, pathogenic/likely pathogenic variants were the least common and included two variants in three patients.

Calculating minor allele frequency and plotting graphs thereof, we noted that minor allele frequencies for all variants, except for BRCA1 c.4987-92A>G(rs8176233), BRCA1 c.3113A>G(rs16941), BRCA1 c.3548A>G(rs16942), and BRCA2 c.2971A>G(rs1799944), had *P* values of 0.05 or less: all four of the variants lacking statistical significance were classified as benign (Supplementary Fig. S1).

In this study, six gene mutations previously classified as VUS were reclassified as likely pathogenic based on ClinVar review. The variants were BRCA1 c.5089T>C (p.Cys1697Arg), BRCA1 c.5509T>C (p. Trp1837Arg), BRCA1 c.5516T>C (p.Leu1839Ser), BRCA1 c.81-9C>G, BRCA2 c.8023A>G (p.Ile2675Val), and BRCA2 c.9004G>A (p.Glu3002Lys).

### Odds ratio estimation

**Figure 2:**
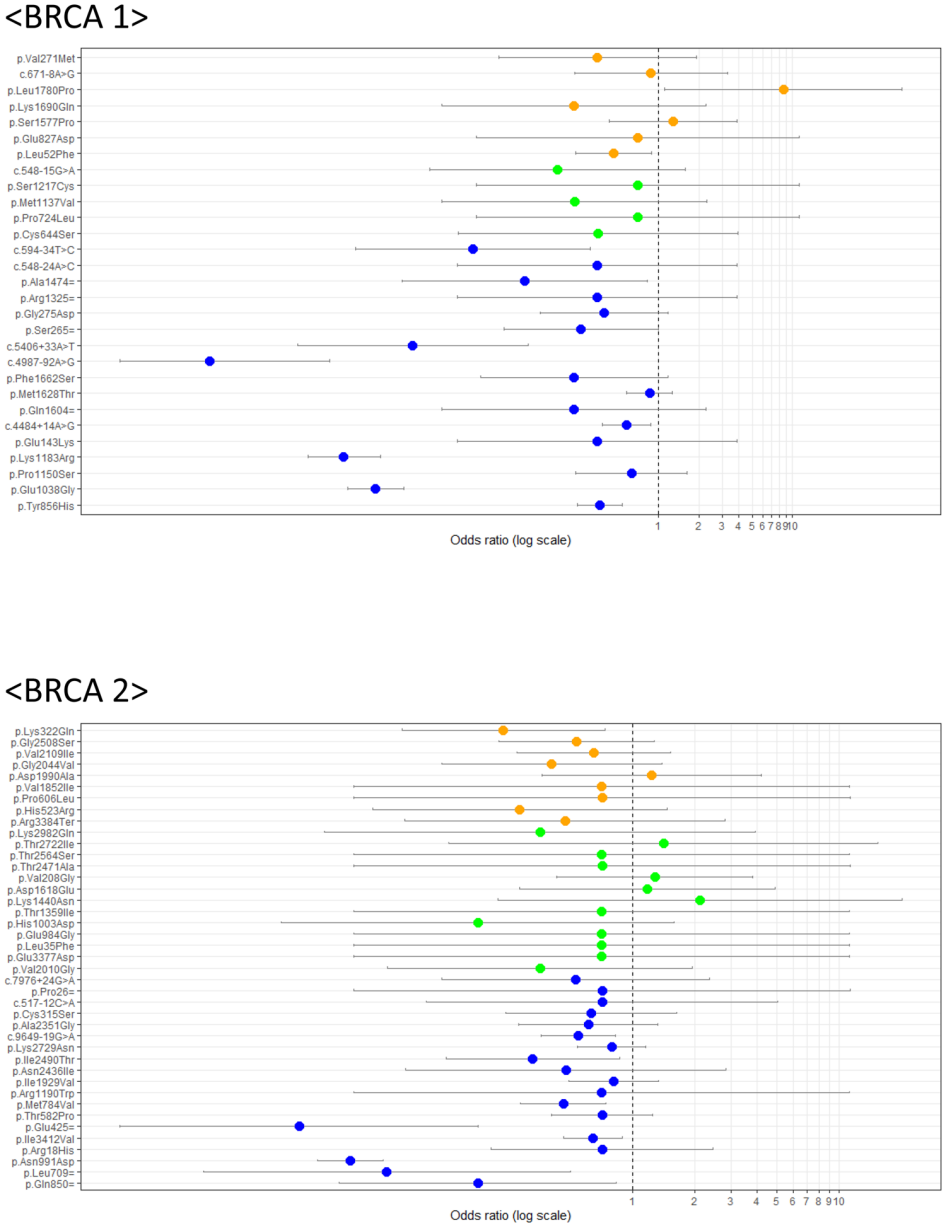
Odds ratios (ORs) using Korean population data from the Korean Reference Genome Database (KRGDB). Variants that could be identified in the KRGDB were classified according to the ClinVar database (vertical axis). Round dots indicate ORs, and the continuous line through each dot indicates a 95% confidence interval. Variants with orange, green, and blue dots indicate conflicting interpretations of pathogenicity, uncertain significance, and benign/likely benign variants according to the ClinVar database, respectively.

We calculated the odds ratios (ORs) for each variant using the KRGDB, as shown in Fig. 2. The OR of BRCA1 c.5339T>C (p.Leu1780Pro) was significantly high in analysis with the Wald method with 95% confidence intervals (CI). The corresponding *P* value was 0.0127 before and 0.889 after Bonferroni correction.

## Discussion

We re-evaluated genetic results in patients with VUSs in *BRCA1* and *BRCA2* using the ClinVar database. Since genetic characteristics can vary by ethnicity^7,9,10^, this study aimed to identify the prevalence of VUS in the Korean population and to re-classify the results of initial genetic tests. Although initial genetic testing results in the KOHBRA study revealed a VUS in 676 patients (278 patients for *BRCA1* and 453 patients for *BRCA2*) out a total of 2,403 breast cancer patients (28.13%, 676/2,403), re-evaluation revealed a lower frequency of VUSs (8.03%, 193/2,403). About a third of the variants that were originally classified as VUS in the KOHBRA study were reclassified as benign/likely benign or pathogenic/likely pathogenic, accounting for two-thirds of all VUS patients (71.45%, 483/676). This result suggests that a re-classification approach using the ClinVar database can reduce the frequency of VUSs in the Korean population.

About two-thirds of the VUSs in the KOHBRA study were reclassified as benign or likely benign [193/278 (69.42%) of *BRCA1* patients, 328/453 (72.41%) of *BRCA2* patients, and 471/676 (69.67%) of all patients]. A third of the mutation types classified as VUS in the KOHBRA study were downgraded to benign or likely benign (20/58 mutations in *BRCA1* and 25/91 in *BRCA2*). These results were consistent with previous studies^1,12,13^: So et al. reported that 30/75 (40%) of VUS patients were reclassified as benign or likely benign^14^. In our study, six patients with *BRCA1* VUS (four mutations) and three patients with *BRCA2* VUS (two mutations) were reclassified as pathogenic or likely pathogenic.

In this study, six mutations in nine patients were reclassified from VUSs to pathogenic or likely pathogenic variants. BRCA1 c.5089T>C (p.Cys1697Arg), BRCA1 c.5509T>C (p. Trp1837Arg), BRCA1 c.5516T>C (p.Leu1839Ser), and BRCA1 c.81-9C>G were interpreted as likely pathogenic among variants in *BRCA1*. BRCA2 c.8023A>G (p.Ile2675Val) and BRCA2 c.9004G>A (p.Glu3002Lys) were interpreted as pathogenic/likely pathogenic among variants in *BRCA2* based on the ClinVar database. Since all six mutations are supported by sufficient evidence in functional studies (PS3)^15–17^, have not been reported in a genomic database for the general Korean population (PM2), are classified as pathogenic in ClinVar (PP5), and are deleterious mutations (PP3) according to an in silico study^18^, they should be reclassified as likely pathogenic or pathogenic mutations. For patients with these mutations, additional genetic counseling and proper management, such as familial genetic testing, risk-reducing medications, or risk-reducing surgery, are needed for the prevention of cancer.

Interestingly, we identified several cases with conflicting interpretations of pathogenicity, for which reviewed the current status of available evidence in the literature (Table 3). In such instances, calculating ORs can help to reclassify them. The KRGDB, which is a large-scale, single-race database collected from 1,722 Koreans, is built for precision medicine research. We analyzed the KRGDB data using the Wald method to obtain ORs and 95% CIs for all VUS variants (Fig. 2). Several mutations (7 *BRCA1* and 9 *BRCA2* mutations) were evaluated. Most of them showed no significance. However, BRCA1 c.5339T>C (p.Leu1780Pro) showed possible pathogenicity, while BRCA1 c.154C>T (p.Leu52Phe) and BRCA2 c.964A>C (p.Lys322Gln) were potentially benign. However, with the most conservative multiple test correction, Bonferroni correction, the statistically significance of c.5339T>C disappeared. This non-significant level should be carefully interpreted, because several previous studies indicated that this c.5339.T>C variant is pathogenic or likely pathogenic according to clinicopathologic features and American College of Medical Genetics (ACMG) guidelines^13,19–21^. Therefore, even though the *P* value after multiple correction indicated a lack of statistical significance, c.5339T>C should be interpreted as a pathogenic or likely pathogenic variant in light of other functional evaluations, including co-segregation, in vitro, and in silico analyses. In verifying additional pathogenic mutation candidates, we merely referred to OR and CI values, but do not insist that OR values alone should be applied. With further accumulation of data in the future, we may be able to recalculate these ORs of *P* values. Meanwhile, variants BRCA 1 c.5014_5016delCAC, BRCA 1 c.5332G>A, BRCA2 c.182T>C, BRCA 2 c.1909 + 22delT, BRCA 2 c.8486A>G, and BRCA2 c.8954-5A>G have not been reported in the general population and should remain classified as VUS, since there are no reports on their deleterious function.

**Table 3:** Conflicting interpretations of pathogenicity.

BRCA1	RS_number	(Likely) Pathogenic	Uncertain significance	(Likely) Benign	No. of patients	OR	CI
c.1357G>C, p.Glu453Gln	rs768054411		3	1	1		
c.154C>T p.Leu52Phe	rs80357084		5	3	15	0.4646	0.2421–0.8914
c.2481A>C p.Glu827Asp	rs397508970		2	1	1	0.7082	0.0446–11.2359
c.2726A>T p.Asn909Ile	rs80357127		8	1	2		
c.4729T>C p.Ser1577Pro	rs80356909		1	7	9	1.2874	0.4314–3.8417
c.5014_5016delCAC p.His1673del	rs80358343	3	4		1		
c.5068A>C p.Lys1690Gln	rs397507239		8	1	1	0.2358	0.0246–2.2601
c.5332G>A p.Asp1778Asn	rs80357112	2	3		1		
c.5339T>C p.Leu1780Pro	rs80357474	4	1		12	8.5181	1.1192–64.8277
c.547 + 14delG c.547 + 14delG	rs273902771		2	4	6		
c.671-8A>G c.671-8A>G	rs80358144		1	4	5	0.8852	0.2385–3.2849
c.811G>A p.Val271Met	rs80357244		1	11	2	0.3537	0.065–1.9254

BRCA2	RS_number	(Likely) Pathogenic	Uncertain significance	(Likely) Benign	No. of patients	OR	CI
c.10150C>T p.Arg3384Ter	rs397507568		1	5	2	0.4717	0.0791–2.8122
c.1568A>G p.His523Arg	rs80358443		2	9	2	0.2855	0.0554–1.4714
c.1817C>T p.Pro606Leu	rs80358469		5	1	1	0.7145	0.0448–11.4046
c.182T>C p.Leu61Pro	rs1555280374		2	1	1		
c.1909 + 22delT	rs276174816		1	7	1		
c.317-10A>G	rs81002824		1	1	1		
c.3256A>G p.Ile1086Val	rs80358571		5	2	3		
c.4599A>C p.Lys1533Asn	rs80358694		3	5	1		
c.5554G>A p.Val1852Ile	rs80358777		4	2	1	0.7078	0.0446–11.225
c.5969A>C p.Asp1990Ala	rs148618542		6	1	7	1.2391	0.3642–4.216
c.6101G>A p.Arg2034His	rs80358849		2	1	1		
c.6131G>T p.Gly2044Val	rs56191579		1	8	4	0.4078	0.1194–1.3933
c.6325G>A p.Val2109Ile	rs79456940		2	10	10	0.6488	0.2754–1.5285
c.7522G>A p.Gly2508Ser	rs80358978		7	6	9	0.5353	0.2254–1.2713
c.8092G>A p.Ala2698Thr	rs80359052		6	4	1		
c.8486A>G p.Gln2829Arg	rs80359100	2	1		1		
c.8954-5A>G	rs886040949	5	1		1		
c.964A>C p.Lys322Gln	rs11571640		3	9	4	0.2375	0.0766–0.7367

Interpretations of VUSs are complex. Functional studies for reclassifying VUS could be a promising approach. Traditionally, VUS interpretation has depended on inductive conclusions based on information of individual patients^22^. However, many potential variants in *BRCA* are present at low variant allele frequencies, with phenotypes that are incompletely penetrant. Findlay et al. reported the application of saturation genome editing (SGE) to measure the functional outcomes of all possible single nucleotide variants (SNVs) in key areas of BRCA1^15^. Functional effects were almost concordant with the established assessments of pathogenicity. Function scores using SGE could help with interpreting the significance of VUSs by providing functional classification and assessment of ambiguous or newly-discovered variants.

The four *BRCA1* mutations (c.5089T>C (p.Cys1697Arg), c.5509T>C (p.Trp1837Arg), c.5516T>C (p.Leu1839Ser), and c.81-9C>G) identified as likely pathogenic using the ClinVar database were identified as non-functional in Findlay’s study^15^. The c.5339T>C (p.Leu1780Pro) variant identified as having conflicting interpretations of pathogenicity in the ClinVar database was also identified as “non-functional” in the functional study results, suggesting this variant as pathogenic^15^. On the other hand, other variants with conflicting interpretations of pathogenicity in the ClinVar database (including c.154C>T (p.Leu52Phe), c.5068A>C (p.Lys1690Gln), and c.5332G>A (p.Asp1778Asn)) have been categorized as functional or intermediate^15^. The other *BRCA1* variants with conflicting interpretations of pathogenicity in the ClinVar database could not be evaluated according to Findlay’s study, as it analyzed only RING and BRCT domains as targets^15^.

Cosegregation analysis may also be helpful in re-evaluating VUSs. Zuntini et al*.* performed cosegregation analysis for 13 VUSs in 11 kindreds to improve VUS evaluation, and two variants were found to have additional supporting evidence of pathogenicity^23^. Among the variants that were reclassified as pathogenic variants in our study, BRCA1 c.5509T>C (p. Trp1837Arg) was discussed in Zuntini's study as well; however, due to the distinct nature of our data, cosegregation analysis could not be conducted. Cosegregation analysis may help improve understanding of VUSs and provide genetic counseling for specific families, sufficient for pedigree analysis.

When ORs were calculated using the KRGDB for all KOHBRA data, the OR of BRCA1 c.5339T>C (p.Leu1780Pro) was found to be significantly elevated (Fig. 2). This variant was also identified as non-functional in Findlay’s study. In addition, several studies have suggested that this mutation is pathogenic based on other evidence, including a strong family history of breast and ovarian cancer, absence in general population data, impaired function demonstrated by in silico studies, and triple negativity in clinicopathologic features^13,14,21^. Previous studies have used a similar approach to reclassify some variants^24,25^.

One limitation of the study is that we reviewed the VUSs by assigning them to a database based on a mostly Caucasian population. Researchers contributing to the Single Nucleotide Polymorphism (SNP) database or ClinVar tend to be concentrated in Western countries, leading to a lack of registration of major variants in Asian populations or a lack of interpretation of variants such as L1780P. Nevertheless, this study was meaningful in that it confirmed VUS status in Koreans using a prospective study and lays the groundwork for broadening our understanding of VUSs and conducting further research. Another limitation of the study is that there is missing information in the KOHBRA data, which are necessary to reclassify VUSs (e.g., 27 variants were not submitted to the SNP database). However, the lacking data comprised only 4.29% of the cohort and would unlikely weaken the power of the current study.

Taken together, most of the mutations that were classified as VUS in the KOHBRA study were reclassified as benign. Four VUSs in *BRCA1* and two in *BRCA2* VUS were reclassified as pathogenic or likely pathogenic. When ORs were calculated using the KRGDB for all KOHBRA data, the OR of BRCA1 c.5339T>C (p.Leu1780Pro) was significantly high, although ClinVar considered BRCA1 c.5339T>C to have conflicting interpretations of pathogenicity. These seven mutations could be reclassified as likely pathogenic or pathogenic mutations, according to ACMG guidelines. Since the mutations classified as benign in ClinVar have a high normal frequency, it is desirable to judge them as benign.

However, some VUSs remained as having conflicting interpretations of pathogenicity, rather than being re-assessed as benign or pathogenic. Their characteristics will likely be more discernable with the accumulation of more information. When a VUS is reclassified as pathogenic/likely pathogenic, appropriate management, including risk-reducing medication and surgery, should be discussed with patients and their families. In addition to collecting individual data, functional studies using genetic techniques, such as SGE, could help contribute to the functional classification and assessment of VUSs.

## Methods

### Subjects

The study population was obtained from the Korean Hereditary Breast Cancer (KOHBRA) study^26^. The KOHBRA study is a multicenter prospective cohort study designed to investigate the prevalence and causes of hereditary breast cancer in the Korean population. Through the study, 3,015 subjects were recruited between May 2007 and December 2013 from 36 institutions^27^. The eligibility criteria were as follows: (1) breast cancer patients with a family history of breast or ovarian cancer; (2) breast cancer patients without a family history of breast or ovarian cancer (non-familial) who were aged 40 years or younger at diagnosis and were diagnosed with bilateral breast cancer or another primary malignancy; (3) male breast cancer patients; and (4) family members of *BRCA 1/2* mutation carriers. After excluding several subjects, a total of 2953 subjects (1228 familial breast cancer patients, 1175 non-familial breast cancer patients, and 550 family members of affected carriers) were evaluated. We identified 676 breast cancer patients with VUS on BRCA mutation tests.

These results were reclassified using the ClinVar database (http://www.ncbi.nlm.nih.gov/clinvar/) based on refSNP (RS) numbers. Odds ratios (ORs) for each variant were calculated using Korean population data from the KRGDB, which was established by conducting whole genome sequencing of 1,722 Koreans^28^. Variants that were not registered in the Single Nucleotide Polymorphism (SNP) database are shown in Table 1. In this study, variations without RS numbers were also included in the denominator when checking the overall frequency of VUS.

### *BRCA 1/2* mutation analysis

*BRCA 1/2* genetic testing was performed using genomic DNA from the peripheral blood. Of the 2403 patients, clinicians used fluorescence confirmation sensitive capillary (gel) electrophoresis on 1183 patients, direct sequencing on 1101 patients, and denaturing high-performance liquid chromatography on 119 patients^27^. Each testing method was selected according to the laboratory linked to the institution. All BRCA 1/2 mutations were classified as pathogenic, VUS, or polymorphic.

### Statistical analysis

Chi-square or Fisher’s exact tests were used for categorical variables. ORs and 95% confidence intervals were obtained using the Wald method. We retrieved allele frequencies from 1722 Koreans (KRGDB). For each variant, an OR was calculated based on its occurrence in 2403 patient cases and in the KRGDB. All analyses were performed using SPSS version 23 (SPSS; Chicago, IL, USA), and statistical significance was defined as *P* < 0.05.

### Mutation nomenclature

All sequence variations are described according to the HUGO-approved systematic nomenclature (http://www.hgvs.org/mutnomen/) using GenBank reference sequences (NM_007294.2 for *BRCA 1* and NM_000059.3 for *BRCA 2*). The breast cancer information core nomenclature is also presented for convenience.

This study was approved by the Institutional Review Board of Severance Hospital, Yonsei University. (IRB# 4-2017-0255).

## Supplementary information

Supplementary information

## Data Availability

The genotype and clinical phenotype data that support the findings of this study are not publicly available due to ethical and patient consent constraints. However, genotype and basic clinical phenotype data are available upon reasonable request from the corresponding author [H.S.P.] under a collaboration and data usage agreement.
