# Author Correction: Analysis of BRCA1/2 variants of unknown significance in the prospective Korean Hereditary Breast Cancer study

**DOI:** 10.1038/s41598-023-31093-x

**Published:** 2023-03-08

**Authors:** Joo Heung Kim, Sunggyun Park, Hyung Seok Park, Ji Soo Park, Seung‑Tae Lee, Sung‑Won Kim, Jong Won Lee, Min Hyuk Lee, Sue K. Park, Woo‑Chul Noh, Doo Ho Choi, Wonshik Han, Sung Hoo Jung

**Affiliations:** 1 Department of Surgery, Yongin Severance Hospital, Yonsei University College of Medicine, Yongin, Gyeonggi Republic of Korea; 2 Department of Laboratory Medicine, Keimyung University School of Medicine, Daegu, Republic of Korea; 3 Department of Surgery, Yonsei University College of Medicine, 50‑1 Yonseiro, Seodaemun‑gu, Seoul, 03722 Republic of Korea; 4 Hereditary Cancer Clinic, Cancer Prevention Center, Yonsei Cancer Center, Yonsei University College of Medicine, 50‑1 Yonseiro, Seodaemun‑gu, Seoul, 03722 Republic of Korea; 5 Department of Laboratory Medicine, Yonsei University College of Medicine, Seoul, Republic of Korea; 6 Department of Surgery, Daerim St. Mary’s Hospital, Seoul, Republic of Korea; 7 Department of Surgery, Asan Medical Center, University of Ulsan College of Medicine, Seoul, Republic of Korea; 8 Department of Surgery, Soonchunhyang University Seoul Hospital, Seoul, Republic of Korea; 9 Department of Preventive Medicine, Seoul National University College of Medicine, Seoul, Republic of Korea; 10 Cancer Research Institute, Seoul National University, Seoul, Republic of Korea; 11 Department of Surgery, Korea Institute of Radiological & Medical Science, Korea Cancer Center Hospital, Seoul, Republic of Korea; 12 Department of Radiation Oncology, Samsung Medical Center, Sungkyunkwan University, Seoul, Republic of Korea; 13 Department of Surgery, Cancer Research Institute, Seoul National University College of Medicine, Seoul, Republic of Korea; 14 Department of Surgery, Chonbuk National University Hospital, Jeonju, Jeollabuk Republic of Korea

Correction to: *Scientific Reports* 10.1038/s41598-021-87792-w, published online 19 April 2021

The Funding section in the original version of this Article was omitted. The Funding section now reads:

“The present research has been supported by Korea Breast Cancer Foundation (KBCF-2016U002).”

The original Article has been corrected.

