# Supplementary material for: Analysis of BRCA1/2 variants of unknown significance in the prospective Korean Hereditary Breast Cancer study
Source: Sci Rep. 2021 Apr 19;11:8485. doi: 10.1038/s41598-021-87792-w (PMC8055990; doi:10.1038/s41598-021-87792-w)

**-Title page-**

**Title:** Analysis of BRCA1/2 variants of unknown significance in patients with breast cancer from a prospective KOHBRA study

**Authors:**

Joo Heung Kim^1*^, Sunggyun Park^2*^, Hyung Seok Park^3^, Ji Soo Park^4^, Seung-Tae Lee^5^, Sung-Won Kim^6^, Jong Won Lee^7^, Min Hyuk Lee^8^, Sue K. Park^9,10^, Woo-Chul Noh^11^, Doo Ho Choi^12^, Wonshik Han^13^, Sung Hoo Jung^14^

**Affiliations:**

^1^Department of Surgery, Yongin Severance Hospital, Yonsei University College of Medicine, Yongin, Gyeonggi, Republic of Korea

^2^Department of Laboratory Medicine, Keimyung University School of Medicine, Daegu, Republic of Korea

^3^Department of Surgery, Yonsei University College of Medicine, Seoul, Republic of Korea

^4^Hereditary Cancer Clinic, Cancer Prevention Center, Yonsei Cancer Center, Yonsei University College of Medicine, Seoul, Republic of Korea

^5^Department of Laboratory Medicine, Yonsei University College of Medicine, Seoul, Republic of Korea

^6^Department of Surgery, Daerim St. Mary’s Hospital, Seoul, Republic of Korea

^7^Department of Surgery, Asan Medical Center, University of Ulsan College of Medicine, Seoul, Republic of Korea

^8^Department of Surgery, Soonchunhyang University Seoul Hospital, Seoul, Republic of Korea

^9^Department of Preventive Medicine, Seoul National University College of Medicine, Seoul, Republic of Korea

^10^Cancer Research Institute, Seoul National University, Seoul, Republic of Korea

^11^Department of Surgery, Korea Institute of Radiological & Medical Science, Korea Cancer Center Hospital, Seoul, Republic of Korea

^12^Department of Radiation Oncology, Samsung Medical Center, Sungkyunkwan University, Seoul, Republic of Korea

^13^Department of Surgery, Cancer Research Institute, Seoul National University College of Medicine, Seoul, Republic of Korea

^14^Department of Surgery, Chonbuk National University Hospital, Jeonju, Jeollabuk, Republic of Korea

**Co-correspondence:**

Hyung Seok Park, MD, PhD

Department of Surgery, Yonsei University College of Medicine

50-1 Yonseiro, Seodaemun-gu, Seoul 03722, Republic of Korea

Tel: +82-2-2228-2100, Fax: +82-2-323-8289

E-mail: imgenius@yuhs.ac

Ji Soo Park, MD, PhD

Hereditary Cancer Clinic, Cancer Prevention Center, Yonsei Cancer Center, Yonsei University College of Medicine

50-1 Yonseiro, Seodaemun-gu, Seoul 03722, Republic of Korea

Tel: +82-2228-4940

E-mail: [PMJISU@yuhs.ac](mailto:PMJISU@yuhs.ac)

*Both authors contributed equally to this manuscript.

Supplementary Fig. S1 Minor allele frequency (MAF) based on the KRGDB

BRCA1


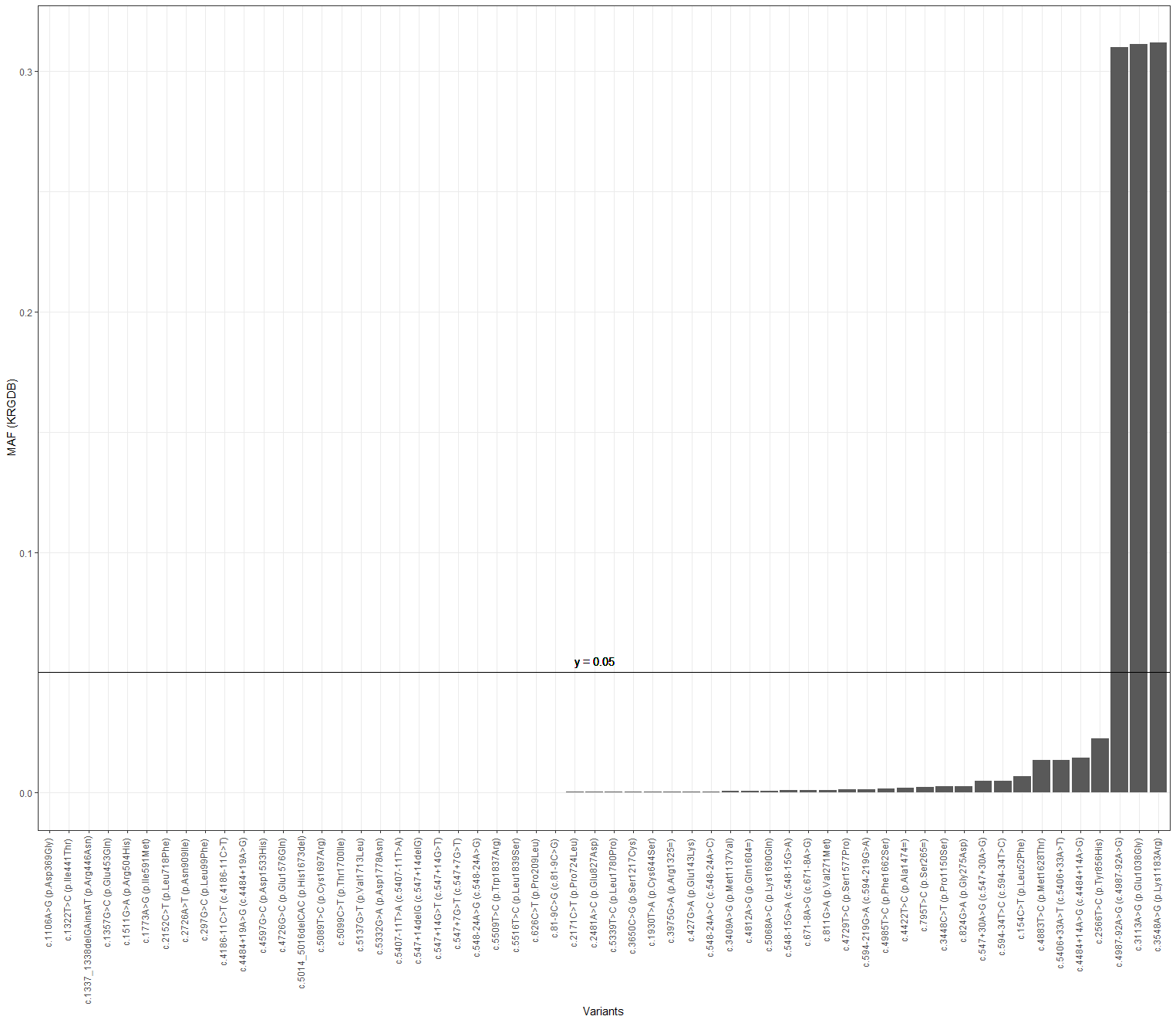


BRCA2


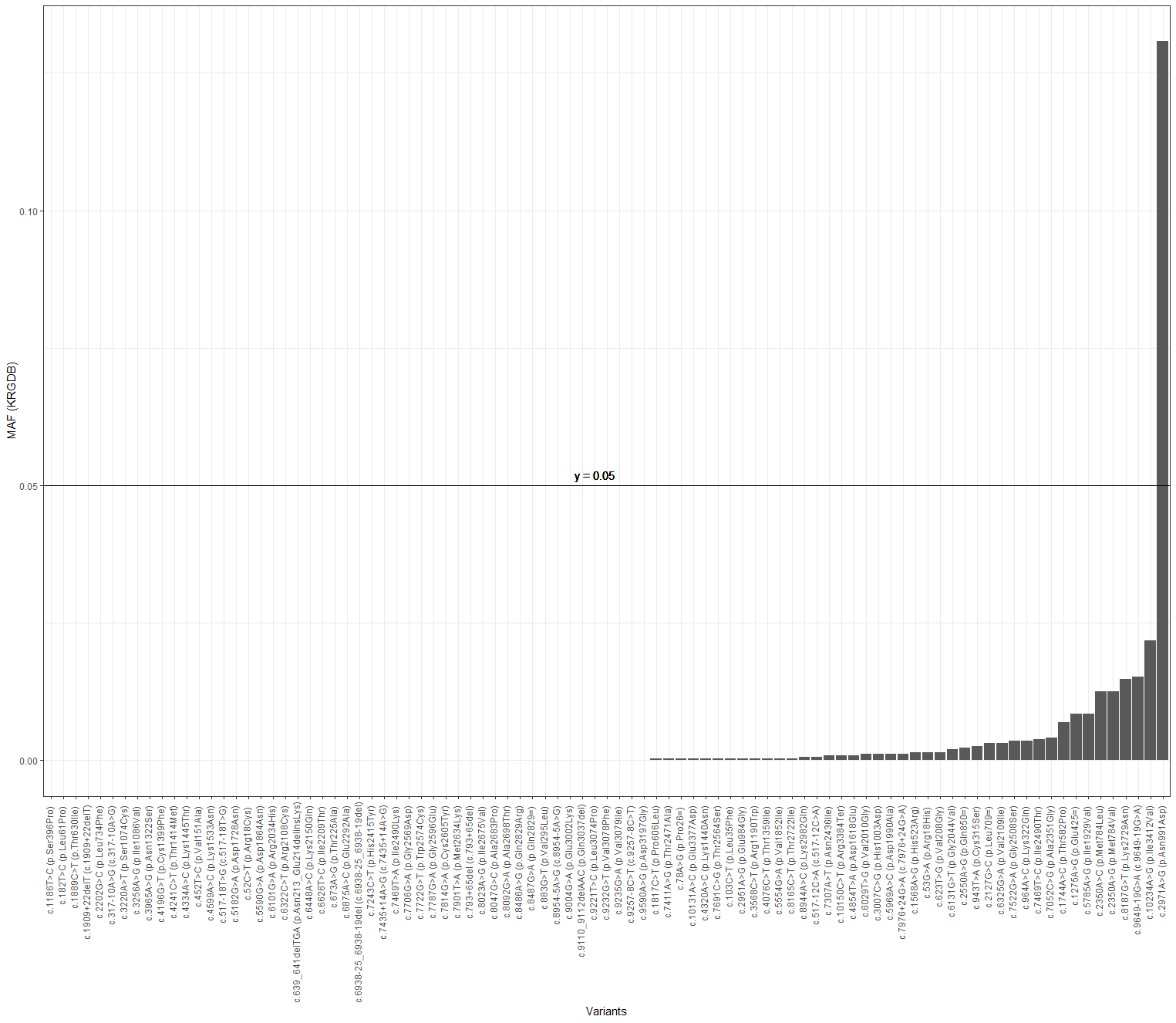

Supplement: Supplementary file 1 — Supplementary information [file 41598_2021_87792_MOESM1_ESM.docx]
